# Does a 12-Month Transitional Care Model Intervention by Geriatric-Experienced Care Professionals Improve Nutritional Status of Older Patients after Hospital Discharge? A Randomized Controlled Trial

**DOI:** 10.3390/nu13093023

**Published:** 2021-08-29

**Authors:** Neshat Chareh, Anja Rappl, Martina Rimmele, Klaus Wingenfeld, Ellen Freiberger, Cornel C. Sieber, Dorothee Volkert

**Affiliations:** 1 Institute for Biomedicine of Aging, Friedrich-Alexander-Universität Erlangen-Nürnberg, 90408 Nuremberg, Germany; merimmele@yahoo.com (M.R.); ellen.freiberger@fau.de (E.F.); cornel.sieber@fau.de (C.C.S.); dorothee.volkert@fau.de (D.V.); 2 Institute for Medical Informatics, Biometry, and Epidemiology, Friedrich-Alexander-Universität Erlangen-Nürnberg, 91054 Erlangen, Germany; anja.rappl@fau.de; 3 Institute for Nursing Science, University of Bielefeld, 33615 Bielefeld, Germany; Klaus.Wingenfeld@uni-bielefeld.de; 4 Department of Medicine, Kantonsspital Winterthur, 8401 Winterthur, Switzerland

**Keywords:** older adults, nutrition advice, transitional care, hospital discharge, geriatric rehabilitation

## Abstract

At hospital discharge, many older patients are at health and nutritional risk, indicating a requirement for ongoing care. We aim to evaluate the effects of comprehensive individualized care by geriatric-experienced care professionals, the so-called “pathfinders”, on nutritional status (NS) of older patients after discharge. A total of 244 patients (median age 81.0 years) without major cognitive impairment were randomized to Intervention Group (IG: 123) or Control Group (CG: 121) for a 12-month intervention, with up to 7 home visits and 11 phone calls. The comprehensive individualized care contained nutritional advice, when required. The intervention effect after three (T3m) and 12 (T12m) months on change in MNA-SF (Mini Nutritional Assessment-Short Form) and BMI was evaluated by Univariate General Linear Model (ANOVA), adjusted for age, sex, living situation, and activities of daily living. At baseline, mean MNA-SF did not differ between IG and CG (10.7 ± 2.6 vs. 11.2 ± 2.5, *p* = 0.148); however, mean BMI was significantly lower in IG compared to CG (27.2 ± 4.7 vs. 28.8 ± 4.8 kg/m^2^, *p* = 0.012). At T3m, mean change did not differ significantly between the groups, neither in MNA-SF (0.6; 95%CI: −0.1–1.3 vs. 0.4; −0.3–1.1, *p* = 0.708) nor in BMI (−0.2; −0.6–0.1 vs. 0.0; −0.4–0.4 kg/m^2^, *p* = 0.290). At T12m, mean change of MNA-SF was significantly higher in IG than in CG (1.4; 0.5–2.3 vs. 0.0; −0.9–0.8; *p* = 0.012). BMI remained unchanged in IG, whereas it slightly declined in CG (0.0; −0.7–0.6 vs. −0.9; −1.6–−0.2 kg/m^2^, *p* = 0.034). We observed rather small effects of comprehensive individualized care by pathfinders on NS in older patients 12 months after discharge. For more pronounced effects nutrition expertise might be needed.

## 1. Introduction

Malnutrition is a common issue among older adults and prevalence rates of 21% to 45% have been reported according to MNA (Mini Nutritional Assessment) in hospitalized older patients [1,2,3,4,5]. Furthermore, a hospital stay is a critical period, which may deteriorate nutritional status and lead to malnutrition at discharge [6]. Malnutrition in older patients is associated with a higher risk of readmission to hospital, reduced quality of life, increased health care costs, as well as increased risk of morbidity and mortality [5,7,8].

To improve the nutritional status of older adults after hospital discharge, several nutritional intervention studies with different approaches such as dietary counselling, dietary enrichment, or providing oral nutritional supplements have been performed. A 12-week randomized controlled trial with three follow-up home visits after discharge by registered dietitians showed an improvement in body weight, energy, and protein intake of geriatric patients with a median age of 80 years and at risk of malnutrition after three months [9]. Another randomized controlled trial with protein-enriched foods and drinks for patients with a mean age of 77 years for three-month after discharge indicated an enhancement in body weight and nutritional status according to MNA after six months; however, no differences were observed between intervention and control group [10]. A recent review including nine randomized controlled trials assessed the effectiveness of individualized nutritional care plans designed either in the hospital or after discharge in older adults. This review suggested that an improvement in the nutritional status of older adults might be achieved through individualized nutritional care plans by dietitians [11].

However, it is important to consider that malnutrition in older adults is a multifaceted issue and multiple factors such as health problems, functional and cognitive impairments, poor social and economic condition, and polypharmacy may contribute to an increased risk of malnutrition [7,12,13]. Therefore, to support adequate nutrition and improvement of nutritional status after discharge from hospital, a holistic approach seems to be a reasonable approach by focusing on not only nutrition but also other underlying problems. In 2019, Vearing et al. investigated the impact of a 12-week post-hospital transitional program with assistance from a multidisciplinary team including a dietitian on nutritional status in older adults. The study showed an improvement in nutritional status based on MNA in older patients with a mean age of 82 years after hospital discharge; however, this study lacks a control group [3].

Another considerable problem in older hospitalized patients is a lack of continuity in care and poor coordination of care among health care providers after discharge, which may negatively affect rehabilitation and consequently deteriorate nutritional status [8,14,15]. In Germany, in order to assure an appropriate transition process and continuity of care from hospital to home, hospitals are obligated to provide discharge planning shortly before hospital discharge. This is, however, focused on the period immediately after discharge and has shown to be insufficient in everyday clinical practice [16,17]. In 1994, Naylor et al. developed the Transitional Care Model (TCM) to ensure appropriate and adequate care for older patients transitioning from hospital to home [18]. In this model, transitional care is a set of comprehensive individualized care management strategies carried out exclusively by nurse specialists or trained health professionals to coordinate safe and proper care transitions and ensure continuity of care for patients across the care settings, especially from hospital to home [18,19].

Based on TCM [18], a randomized controlled trial was conducted in Germany to improve the care transition from hospital to home for geriatrics patients [20]. The study aimed to achieve a reduction in hospital readmission rate by improving older patients’ care, including improvement of their nutritional situation at home after hospital discharge through a comprehensive individualized care plan by geriatric-experienced care professionals. In this context, the question arose whether this broad approach without nutritional expert but considering nutrition problems can have positive effects on nutritional status. To our knowledge, this has not been evaluated before. Thus, in this present study, we focus on the effects of TCM on nutritional status of older patients and aim to evaluate if this broad approach might be sufficient in achieving improvement in nutritional status.

## 2. Materials and Methods

### 2.1. Trail Design, Registration, and Ethical Approvement

This study is a secondary analysis of the project TIGER (Transsectoral Intervention Program for Improvement of Geriatric Care in Regensburg), which was a 12-month non-blinded randomized controlled trial with two arms. The study was carried out from April 2018 until June 2020 in St John of God hospital (Barmherzige Brüder) in the city of Regensburg and its surrounding area in Germany, and is described in detail elsewhere [20]. The study was registered in ClinicalTrials.gov (Identifier: NCT03513159) and its study protocol was approved by the ethics committee of the Friedrich-Alexander-Universität Erlangen-Nürnberg (FAU) on 5 March 2018 (# 60- 18B). FAU was responsible for the integrity and conduct of the study. The study was conducted according to the Declaration of Helsinki.

### 2.2. Participants

Participants were eligible for the study, (1) if they were 75 years and older, (2) resided within 50 km distance to the hospital in a private household, returned back to their home environment after discharge, (3) were a member of the statutory health insurance AOK (Allgemeine Ortskrankenkasse) Bavaria, and (4) without major cognitive impairment (at least 22 points in Mini Mental State Examination, MMSE), to ensure that they can benefit from the self-management approach of our intervention. Patients who were in palliative situation or had planned readmission to the hospital within the next four weeks were excluded. A TIGER-specific computer-based tool was used to identify potential participants based on the first three eligibility criteria from all wards of the hospital. Thereafter, the staff visited the patients in person and informed them regarding our study. Once each patient signed inform consent, MMSE was performed as the last inclusion criterion for recruitment.

### 2.3. Randomization

Participants were randomly assigned to intervention group (IG) or control group (CG) one day before discharge by using a computer based electronic Data Acquisition and Case Report Form (eCRF) system (secuTrial^®^). Stratified block randomization was performed for gender (male/female), mobility (can walk at least four stair steps—yes/no), and living situation (living alone—yes/no). These three Strata were chosen for randomization, because of their potential effects on the overall need for care. The TIGER statistician (A.R.) generated the random allocation sequence and the randomization blocks varied between 2, 4, and 6. When the answers to the three randomization questions were filled for a participant, the system automatically randomized the participant.

### 2.4. Study Timeline

The initially planned duration of the study for each participant was 12 months. The first contact (T0) took place one day before discharge in hospital. Two home visits were carried out 3 months (T3m) and 12 months (T12m) after discharge. Due to slower recruitment than planned, the recruitment phase was prolonged; however, because of financial and time restrictions, the end of the study duration (June 2020) could not be extended. Therefore, the study period reduced to nine or six months for participants who were recruited after July and after October 2019 [20].

### 2.5. Study Staff

The study was carried out by experienced care professionals (four registered nurses, one case manager, one head nurse, and one occupational therapist) with at least five years of experience in geriatric patient care who acted as the so-called “pathfinders” being responsible for IG or as study nurses for CG. Each participant was accompanied exclusively by one pathfinder or study nurse.

### 2.6. Pathfinders’ Educational Training

Prior to the start of the study, the pathfinders, received an educational program on different aspects of geriatrics care requirements in older adults [20]. Regarding nutrition, all pathfinders received an educational session (one afternoon) about nutrition in older adults from a nutrition scientist specialized in this field with many years of teaching experience (DV) as well as written information material. Potential nutritional risk factors in older adults, signs of nutritional problems, and possible interventions were explained. Pathfinders were instructed to pay attention to nutritional risk factors and initiate adequate actions to tackle these risk factors, e.g., discussion with patient and relatives, initiation of prescription of oral nutritional supplements (ONS) by family physicians or organizing nutritional counselling, or meals on wheels (Table 1).

### 2.7. Intervention

The intervention was designed based on the Transitional Care Model [18,20]. Pathfinders, in collaboration with patients, family members, and care team (e.g., family physicians, hospital care and discharge planning team, ambulant care services) developed a comprehensive individualized care plan for IG patients. To create the care plan, pathfinders arranged the first home visit within the first week after discharge to precisely assess participants’ situation at home. The pathfinders’ assessment was supported by a standardized questionnaire instrument to identify individual care needs at home. The instrument was designed based on various aspects of geriatric care such as participants’ health problems and needs, nutritional problems, physical and cognitive functionality, and medications. Additionally, home environment conditions were evaluated, particularly regarding difficulties in housekeeping and self-care. For each of these aspects, the pathfinders assessed the patient’s need for support and suggested appropriate strategies to eliminate existing problems, e.g., organizing long-term care insurance benefits or day care. Pathfinders actively monitored the situation and adjusted the care plan if required, during home visits as well as phone calls. In the first 3 months after discharge, three home visits and four phone calls were planned. Thereafter, another home visit (6 months after discharge) and one telephone contact per month until T12m were intended. The number of actual home visits and phone calls varied as needed and was adapted according to participants’ preferences and values. It is important to mention, that in implementing the individualized care plan, pathfinders did not provide active care services themselves and did not compete with the activity of usual ambulant services. In fact, their role was advisory and organizational. After evaluating participants’ conditions, they discussed and coordinated the care plan with the participant and his/her care team for the required and desired activities.

### 2.8. Control Group

Before discharge, participants in CG received usual discharge planning from hospital staff including, e.g., provision of medication for the first few days after discharge or first appointment for ensuing therapy [20]. CG did not receive assistance from the TIGER staff, and the two home visits were for the sole purpose of data collection.

### 2.9. Data Collection

Data were assessed after randomization at T0, and then at T3m, and at T12m by pathfinders for IG and by study nurses for CG patients.

#### 2.9.1. Participants’ Characteristics at Baseline

At T0, participants’ date of birth, sex, living situation, and number of medications were extracted from medical reports. Participants’ emotional status was evaluated using Geriatric Depression Scale (GDS; 0–15 points), where a score of 11–15 points indicates a severe depressive mood, 6–10 points a moderate, and 0–5 points a non-depressive mood [21]. The ability to perform basic activities of daily living (Barthel-ADL; 0–100) was assessed according to Mahoney and Barthel [22]. A score below 35 points was defined as severe, 35–60 points as moderate, and 65–100 points as slight limitations [23].

#### 2.9.2. Nutritional Assessment

Nutritional status was determined by the Mini Nutritional Assessment-Short Form (MNA-SF; 0–14 points), which is commonly used and validated for older adults [24], and by Body Mass Index (BMI) at T0, T3m, and T12m. Participants were categorized as well nourished (12–14 points), at risk of malnutrition (8–11 points), or malnourished (0–7 points) [24]. Weight was measured at each time point in light clothing without shoes using a digital scale. Height was measured in hospital without shoes at the most straight standing position using a folding ruler. BMI was calculated as weight (kg)/height (m^2^) and categorized according to the Global Leadership Initiative on Malnutrition (GLIM) where for older adults ≥ 70 years, low BMI is defined as values < 22 kg/m^2^, normal BMI as 22–30 kg/m^2^ and high BMI as ≥30 kg/m^2^ [25].

### 2.10. Outcome Measures

Outcomes in this study were changes in nutritional status between baseline and the three- and 12 months follow-up visits after hospital discharge based on MNA-SF score and BMI.

### 2.11. Power Calculation

The power calculation was based on the primary outcome of the TIGER study, which was the reduction of hospital readmission rate. A reduction of readmission rate within one year of about 25% by the intervention was expected. The significance level was set at 5% and the power at 80% with equal size in the intervention and the control group. Based on the assumption of 15% to 25% of loss-to-follow up, 280 participants were calculated to be adequate. In addition, we applied a post hoc power analysis using the outcome measures from this secondary analysis to determine the necessary sample size. Setting alpha to 0.05, power to 80%, and assuming the change of our means and SD after 12 months are replicated, a total sample size of 124 for MNA-SF and 178 for BMI was calculated.

### 2.12. Statistical Analysis

Results from categorical variables are presented as percentage and from continuous variables as median (P25-P75) (non-normally distributed) or mean (SD) (normally distributed). Chi-square test, Mann–Whitney-U-test and independent sample *t*-test were performed to compare the baseline variables between IG and CG in participants who had available MNA-SF at hospital discharge and in participants who had available MNA-SF at T12m. Intervention effects at T3m and T12m were evaluated by Univariate General Linear Models (using ANOVA). For the 12 months analysis, we excluded participants with the T12m visit after nine and six months, in order to be consistent with the intervention time for all participants. Change in MNA-SF score and change in BMI at T3m from baseline and at T12m from baseline were considered as the dependent variable and all recorded measurements were included in the models. Two different models were calculated, one unadjusted (model 1) and one adjusted (model 2). The adjusted model was controlled for baseline Barthel-ADL, age, living situation, and sex. Furthermore, to test the interaction of treatment with each MNA-SF, as well as BMI category at T3m and at T12m, the categorized MNA-SF and BMI at T0 were added into the respective adjusted and unadjusted model. The estimated marginal means with 95% confidence interval (CI) and their respective *p*-value are reported. A *p*-value < 0.05 was considered as a statistically significant result. Statistical analysis was performed using SPSS version 25.0 (IBM, Munich, Germany).

## 3. Results

### 3.1. Participants’ Flow during the Study

**Figure 1:**
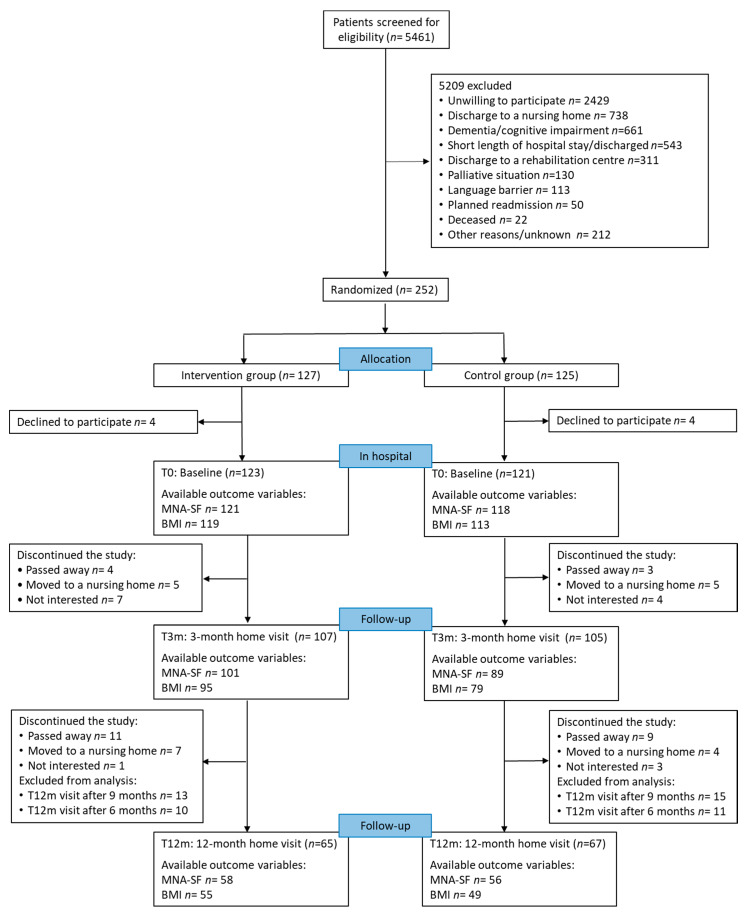
Flow of patient participation during the study, available outcome variables and reasons for dropout and exclusion from analysis; MNA-SF: Mini Nutritional Assessment-Short Form; BMI: Body Mass Index.

Of 5461 screened patients, 252 patients agreed to participate and were randomized. A total of 127 patients were allocated to IG and 125 patients to CG. Four patients in each group withdrew consent within 14 days after signing. Thus, 244 individuals continued the participation. During the first three months, 28 participants discontinued the study. From T3m until T12m, another 35 participants dropped out from the study. Participants’ reasons for withdrawal, the number of excluded participants due to the fact that the final visits were after nine and six months, and the number of available MNA-SF, as well as BMI values at each time point are shown in Figure 1.

### 3.2. Participants’ Characteristics at Hospital Discharge

Participants’ median age was 81.0 years and 56.1% were female. Participants in IG were significantly older compared to participants in CG (Table 2). The vast majority of participants had a non-depressed mood and showed slight limitations in Barthel-ADL Index. Furthermore, among the participants who completed the study, those in IG were significantly older than those in CG (Table 2). No other significant differences were observed between IG and CG in the participants who completed the study. However, those who dropped out from the study were significantly older and more often at risk of malnutrition compared to those who completed the study (Appendix A, Table A1).

### 3.3. Nutritional Status

**Figure 2:**
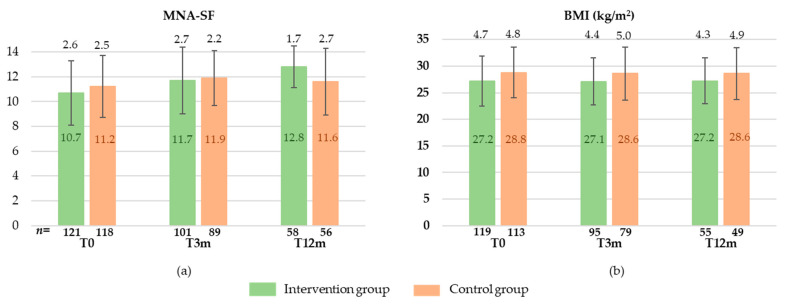
MNA-SF (Mini Nutritional Assessment-Short Form) (**a**) and BMI (Body Mass Index) (**b**) at baseline (T0), after 3 months (T3m), and after 12 months (T12m) in intervention and control group (means ± standard deviation).

At baseline, MNA-SF did not differ between the groups (10.7 ± 2.6 vs. 11.2 ± 2.5; *p* = 0.148). However, BMI was significantly lower in IG than in CG (27.2 ± 4.7 vs. 28.8 ± 4.8 kg/m^2^; *p* = 0.012). MNA-SF scores and BMI values at each time point are provided in Figure 2.

The changes in nutritional status after three and 12 months from baseline in IG and CG are shown in Table 3. After three months, the change in nutritional status did not differ significantly between the groups, neither in MNA-SF nor in BMI, in both unadjusted and adjusted models. Furthermore, the interaction of MNA-SF subgroups and categorized BMI with treatment was not significant. After 12 months, the mean change in MNA-SF in the IG was significantly higher compared to CG in the unadjusted as well as in the adjusted model. Furthermore, the intervention participants maintained their BMI, whereas the control participants reduced their BMI by 0.9 kg/m^2^ during the 12 months of intervention. Although the MNA-SF score improved in participants with malnutrition or at risk of malnutrition and stayed nearly constant in participants with normal nutritional status, the interaction with treatment was not significantly different after 12 months in both adjusted and unadjusted models. Likewise, the interaction of treatment with categorized BMI did not differ significantly.

## 4. Discussion

In this secondary analysis of the TIGER study, we observed a small improvement in nutritional status after one year based on MNA-SF and a prevention of BMI decline in participants receiving individualized support by a pathfinder compared to usual care. Our results indicate a greater improvement in participants with poorer nutritional status at baseline, but this improvement was observed in IG as well as CG.

After three months, the nutritional status regarding MNA-SF improved slightly, without a significant difference between the groups. This improvement in both groups may be explained by a general recovery after the hospital stay. The mean change of BMI after three months was not significantly different between the groups either. These findings are in contrast to earlier studies, where a dietitian was exclusively responsible for the nutrition intervention and an improvement in body weight was found in older patients at nutritional risk after hospital discharge [9,26,27]. In our study, pathfinders provided very general nutritional information (e.g., provision of educational material, recommendation of different recipes high in energy- and protein-dense foods aiming at improving energy and protein intake in case of malnutrition risk, adequate number of meals per day, adequate vegetable, fruit and fluid intake), organized meals on wheels if required or referred to nutritional counselling as part of a comprehensive approach. Thus, the three-month comprehensive individualized care including advice to improve nutrition by a pathfinder may have been too unspecific to improve the nutritional status.

After 12 months, nutritional status based on MNA-SF improved significantly in participants receiving support from a pathfinder compared to those in CG. Regarding BMI, statistically, a deterioration could be prevented in the intervention participants with pathfinders’ support compared to CG. However, considering that the mean of BMI in both groups was above 25 kg/m^2^ and the observed difference between the groups was very small, the clinical relevance of this difference is questionable. Moreover, the weight measurements were performed at participants’ home at different times of the day and in different clothes implying measurement inaccuracies, which may be similar or even greater than the observed difference. Furthermore, our post hoc power calculation indicates that our sample size might be too small to detect a pronounced difference between the groups. In agreement with our finding, an improvement in MNA score has been reported in a six-month randomized controlled trial with individualized nutritional treatment from a dietitian in older adults after hospital discharge; however, no statistically significant difference was observed in body weight there [28]. We are not aware of any other study in this field with an intervention duration of 12 months.

Unlike other studies, where all participants received a nutrition intervention, the individualized care plan in our study addressed a variety of aspects based on each participant’s needs, conditions, and desires and not all participants received necessarily a nutritional recommendation. Furthermore, the content of the plan was adapted at each visit and phone call, based on potential changes in the situation of the participants regarding general health, physical, and social problems. For instance, if participants had difficulties in self-care or household activities, pathfinders organized professional support where, for example, also breakfast or food shopping could be provided if required. Thus, we assume that other non-nutrition-related pathfinder activities within the framework of TCM might indirectly improve the nutritional status in the longer period. One of the important aspects of TCM is the continuity of care by nurses specialized in geriatrics care who organize the hospital discharge plan [18]. Pathfinders provided continuous contacts, home visits, as well as telephone calls for participants in the intervention group, which could encourage and stimulate them to adhere to the recommendations. Moreover, in our study, pathfinders were in close collaboration with participants’ family members, physicians, and caregivers in order to address the complex care needs of older persons and provide adequate care, which might have contributed to the observed effects after a rather long period of 12 months.

However, the intervention effects in our study were very small and the subgroup analysis showed no significant differences between the groups. As pathfinders carried out the intervention, as well as the assessments, it might bring the possibility of bias in performing the data collection (reporting bias). In addition, we also observed that some participants with risk of malnutrition did not receive nutritional advice, which could be due to the fact that none of pathfinders had nutritional background and the necessary knowledge to provide an efficient nutritional support for older adults. This indicates that integrating nutrition expertise in the team might be required for more pronounced effects on nutritional status, which is an essential aspect for older adults’ general health status.

### Strengths and Limitations

One of the strengths of this study is that the intervention was very comprehensive and individualized and was performed in combination of home visits and telephone calls in a rather intense manner. Furthermore, this study had a longer follow-up time compared to other studies. The main limitation is the high loss to follow up of the originally included participants, mainly due to nursing home admission and mortality, which are unfortunately typical problems in this population. Moreover, the intensity of the intervention overall and also of the nutritional intervention varied widely due to the individual problems and arrangements between pathfinders and patients and could consequently not be considered in our analyses. Besides, due to the broad TIGER approach, our sample was not restricted to malnourished persons which diluted the intervention effect. Additionally, based on the study design, which was a nurse-led intervention, a dietician could not be considered in the team for provision of an appropriate nutritional intervention. Finally, MNA-SF is a screening tool, which consists six questions related to nutritional and health status. The tool’s components are obscured by the total score and the origin of change cannot be tracked.

## 5. Conclusions

We observed rather small effects of comprehensive individualized care by pathfinders on nutritional status of patients aged 75 years and older 12 months after hospital discharge. Further studies with the concept of multidisciplinary care for older adults after hospital discharge should consider the fact that addressing each specific aspect of care may need its corresponding expertise in the team. With regard to nutritional care, an expert in nutrition is better able to recognize early signs or the existing nutritional problems and implement more effective measures for improvement of nutritional status in older adults.

## Figures and Tables

**Table 1:** Signs of nutritional problems and possible interventions by pathfinders.

Signs of Nutritional Problems	Possible Interventions
Weight loss Loss of appetite Low food intake Unbalanced diet Impaired taste and/or smell sensation	Provision of educational material (e.g. info-flyer regarding nutrition in older adults) Provision of general nutritional advice (e.g., energy and protein dense foods, size and frequency of meals) Recommendation of oral nutritional supplements (ONS) Referral to nutritional counselling Referral to general practitioner to clarify causes, prescribe ONS
Chewing problem	Referral to dental treatment Provision of recipes for consistency-adapted food
Swallowing problem	Organizing logopedics therapy Checking body and head position during meals Recommendation of consistency-adapted food
Dry mouth	Checking medications for possible side effects Tips on how to ensuring sufficient fluid intake Tips on moistening of the mucous membranes with mouth gel
Lonesome at mealtime Difficulties in preparing meals	Referral to appropriate support services (e.g., shopping assistance, shared meals) Organizing meals on wheels
Nausea and vomiting Abdominal pain Diarrhea or constipation	Referral to general practitioner/family physician

**Table 2:** Baseline characteristics and nutritional status of all participants and the subgroup of those who completed the 12-month follow-up.

Variables		All Participants			Completed the 12-Month		
		IG (121)	CG (118)	*p*	IG (58)	CG (56)	*p*
Age (years), median (P25–P75)		82.0 (79.0–85.0)	80.0 (77.7–84.0)	0.042	81.5 (78.0–85.2)	80.0 (77.0–82.7)	0.031
Female, %		55.4	56.8	0.826	55.2	64.3	0.321
Living situation: alone, %		75.2	75.4	0.969	79.3	76.8	0.745
GDS, %	Severely depressed	1.7	0.8	0.452	3.4	1.8	0.680
	Moderately depressed	14.0	9.3		10.3	14.6	
	No depression	82.6	87.3		84.5	82.2	
	Missing	1.7	2.5		1.7	1.8	
Barthel-ADL, %	Severe limitation	0.8	2.5	0.643	0.0	1.8	0.322
	Moderate limitation	10.7	9.3		12.1	5.4	
	Slight limitation	87.6	88.1		87.9	92.9	
	Missing	0.8	0.0		0.0	0.0	
Number of medications/day, (*n*) median (P25–P75)		(111) 9.0 (6.0–11.0)	(115) 8.0 (5.0–11.0)	0.158	(53) 8.0 (6.0–10.0)	(56) 7.0 (5.0–12.0)	0.865
BMI, %	Low (<22 kg/m^2^)	10.7	5.1	0.182	8.6	5.4	0.196
	Normal (22–30 kg/m^2^)	62.8	59.3		67.2	51.8	
	High (≥30 kg/m^2^)	24.8	31.4		24.1	37.5	
	Missing	1.7	4.2		0.0	5.4	
MNA-SF, %	Malnutrition	13.2	11.9	0.704	12.1	16.1	0.798
	At risk of malnutrition	40.5	36.4		37.9	33.9	
	Well-nourished	46.3	51.7		50.0	50.0	

Number of participants stands for participants with available MNA-SF (Mini Nutritional Assessment-Short Form) at hospital discharge and at T12m; IG: Intervention Group; CG: Control Group; GDS: Geriatric Depression Scale; Barthel-ADL: Activity of Daily Living; BMI: Body Mass Index; Comparing the groups: chi-square test for categorical data, Mann–Whitney-U-test for continuous data.

**Table 3:** Change in nutritional status after 3 and 12 months of intervention from baseline in the whole group and in baseline nutritional status categories ((number of participants) Estimated marginal means with 95% confidence intervals).

		Change from Baseline to T3m		*p*	Change from Baseline to T12m		*p*
		IG	CG		IG	CG	
MNA-SF	Total group	(101) 0.8	(89) 0.7	0.783	(58) 1.9	(56) 0.5	0.017
Model 1		(0.3–1.4)	(0.1–1.4)		(1.2–2.6)	(−0.4–1.4)	
	Malnourished	(12) 4.3	(11) 4.4		(7) 6.0	(9) 4.4	
		(2.9–5.7)	(2.9–5.8)		(4.2–7.7)	(2.9–6.0)	
	At risk of malnutrition	(39) 1.6	(33) 1.8	0.645	(22) 2.6	(19) 1.3	0.921
		(0.8–2.3)	(1.0–2.7)		(1.6–3.8)	(0.2–2.4)	
	Well-nourished	(50) −0.5	(45) −1.0		(29) 0.4	(28) −1.3	
		(−1.2–0.1)	(−1.7–−0.2)		(−0.5–1.2)	(−2.2–−0.4)	
MNA-SF	Total group	(100) 0.6	(89) 0.4	0.708	(58) 1.4	(56) 0.0	0.012
Model 2		(−0.1–1.3)	(−0.3–1.1)		(0.5–2.3)	(−0.9–0.8)	
	Malnourished	(12) 4.4	(11) 4.3		(7) 5.4	(9) 4.1	
		(2.9–5.8)	(2.8–5.9)		(3.8–7.1)	(2.6–5.6)	
	At risk of malnutrition	(39) 1.5	(33) 1.9	0.529	(22) 2.4	(19) 1.0	0.807
		(0.7–2.3)	(1.0–2.7)		(1.3–3.2)	(0.0–2.0)	
	Well-nourished	(49) −0.8	(45) −1.3		(29) −0.2	(28) −1.9	
		(−1.5–−0.1)	(−2.1–−0.7)		(−1.0–0.7)	(−2.8–−1.1)	
BMI	Total value	(95) −0.3	(79) 0.0	0.233	(55) 0.1	(49) −0.7	0.058
Model 1		(−0.6–0.0)	(−0.4–0.4)		(−0.4–0.6)	(−1.3–0.0)	
	Low (<22 kg/m^2^)	(9) 0.5	(5) 0.7		(5) 2.0	(3) 2.0	
		(−0.5–1.5)	(−0.7–2.0)		(0.2–4.3)	(−0.2–4.3)	
	Normal (22–30)	(60) −0.2	(49) −0.2	0.297	(36) 0.2	(26) −0.6	0.815
		(−0.6–0.2)	(−0.6–0.3)		(−0.5–0.8)	(−1.4–0.2)	
	High (≥30)	(26) −0.7	(25) 0.2		(14) −0.8	(20) −1.2	
		(−1.3–−0.1)	(−0.4–0.8)		(−1.8–0.3)	(−2.1–−0.3)	
BMI	Total value	(94) −0.2	(79) 0.0	0.29	(55) 0.0	(49) −0.9	0.034
Model 2		(−0.6–0.1)	(−0.4–0.4)		(−0.7–0.6)	(−1.6–−0.2)	
	Low (<22 kg/m^2^)	(9) 0.6	(5) 0.7		(5) 2.1	(3) 1.6	
		(−0.4–1.6)	(−0.7–2.1)		(0.3–3.9)	(−0.7–3.9)	
	Normal (22–30)	(60) −0.2	(49) −0.2	0.305	(36) 0.0	(26) −0.8	0.896
		(−0.6–0.2)	(−0.6–0.3)		(−0.7–0.7)	(−1.6–0.0)	
	High (≥30)	(25) −0.7	(25) −0.1		(14) −1.0	(20) −1.4	
		(−1.4–−0.1)	(−0.5–0.8)		(−2.2–0.2)	(−2.4–−0.5)	

Univariate General Linear Model; P value stands for interaction of treatment in the whole group and in baseline nutritional status categories; IG: Intervention Group; CG: Control Group; CI: Confidence Interval; MNA-SF: Mini Nutritional Assessment; BMI: Body Mass Index; Model 1: unadjusted; Model 2: adjusted for age, sex, living situation and Barthel-ADL (Activity of Daily Living).

## Data Availability

The data presented in this study are available on request from the TIGER consortium.

## Appendix A

**Table A1:** Baseline characteristics of participants at baseline (T0) for those who completed the study vs. those who dropped out from the study.

		Not Drop-Out (181)	Drop-Out (63)	*p*
Age (years), median (P25–P75)		80.0 (78.0–84.0)	84.0 (79.0–89.0)	0.001
Female, %		58.0	50.8	0.320
Living situation: alone, %		74.6	74.6	0.998
GDS, %	Severely depressed	1.7	0.0	0.283
	Moderately depressed	14.0	15.9	
	No depression	82.6	74.6	
	Missing	1.7	9.5	
Barthel-ADL, %	Severe limitation	1.7	3.2	0.214
	Moderate limitation	9.9	12.7	
	Slight limitation	86.7	77.8	
	Missing	1.7	6.3	
Number of medications/day [*n*] Median (P25–P75)		(175) 8.0 (5.0–11.0)	(53) 9.0 (6.0–12.0)	0.398
BMI, %	Low (<22 kg/m^2^)	7.2	9.5	0.063
	Normal (22–30 kg/m^2^)	56.9	68.3	
	High (≥30 kg/m^2^)	31.5	15.9	
	Missing	4.4	6.3	
MNA-SF, %	Malnutrition	12.7	11.1	0.013
	At risk of malnutrition	33.1	50.8	
	Well-nourished	53.6	31.7	
	Missing	0.0	6.3	

IG: Intervention Group; CG: Control Group; GDS: Geriatric Depression Scale; Barthel-ADL: Activity of Daily Living; BMI: Body Mass Index; MNA-SF: Mini Nutritional Assessment-Short Form; comparing the groups: chi-square test for categorical data, Mann–Whitney-U-test for continuous data.
